# Application of Real-Time 3D Navigation System in CT-Guided Percutaneous Interventional Procedures: A Feasibility Study

**DOI:** 10.1155/2017/3151694

**Published:** 2017-10-18

**Authors:** Priya Bhattacharji, William Moore

**Affiliations:** ^1^Department of Radiology, State University of New York at Stony Brook University Hospital, HSC Level IV, Room 120, Stony Brook, NY 11794, USA; ^2^Department of Radiology, New York University Medical Center, 650 First Avenue, Third Floor, Room 355, New York, NY 10016, USA

## Abstract

**Introduction:**

To evaluate the accuracy of a quantitative 3D navigation system for CT-guided interventional procedures in a two-part study.

**Materials and Methods:**

Twenty-two procedures were performed in abdominal and thoracic phantoms. Accuracies of the 3D anatomy map registration and navigation were evaluated. Time used for the navigated procedures was recorded. In the IRB approved clinical evaluation, 21 patients scheduled for CT-guided thoracic and hepatic biopsy and ablations were recruited. CT-guided procedures were performed without following the 3D navigation display. Accuracy of navigation as well as workflow fitness of the system was evaluated.

**Results:**

In phantoms, the average 3D anatomy map registration error was 1.79 mm. The average navigated needle placement accuracy for one-pass and two-pass procedures, respectively, was 2.0 ± 0.7 mm and 2.8 ± 1.1 mm in the liver and 2.7 ± 1.7 mm and 3.0 ± 1.4 mm in the lung. The average accuracy of the 3D navigation system in human subjects was 4.6 mm ± 3.1 for all procedures. The system fits the existing workflow of CT-guided interventions with minimum impact.

**Conclusion:**

A 3D navigation system can be performed along the existing workflow and has the potential to navigate precision needle placement in CT-guided interventional procedures.

## 1. Introduction

Precision placement of intervention instruments is critical for all procedures especially in percutaneous procedures such as biopsies and ablations in order to achieve diagnostic accuracy as well as accurate tumor targeting. Additionally, with the increasing importance of immune-histochemical markers and molecular markers in cancer patients, the need for larger and more accurate biopsy has become paramount [1, 2]. More recently, the use of minimally invasive treatments that require highly precise imaging guidance such as radiofrequency ablation (RFA), cryoablation, and microwave ablation (MWA) has become common place in cancer management because of their effectiveness and safety [3, 4].

Lesions in soft tissue organs, such as the liver and lung, present a unique challenge for radiologists to target in image guided interventions because of motion and deformation. The modality chosen for these procedures depends on the target of interest, its size, accessibility, and visibility all of which play a crucial role in increasing the complexity of diagnostic and therapeutic interventions.

Computed tomography (CT) and ultrasound (US) are the most commonly used modalities for interventional procedures in the liver and lung. US is of limited utility in the lung and can potentially be limited in the abdominal soft tissues specifically in the ability to detect small lesions, less than 1 cm in diameter [5]. Despite having high accuracy rates [6–8], CT is limited by the lack of real-time imaging which is often necessary for procedural guidance. CT fluoroscopy can result in improvement in needle/probe positioning at the cost of additional radiation exposure to the patient and operator. Additionally, CT fluoroscopy is still a subject of debate for small lesions and lesions near vital anatomies [9, 10]. Cone Beam Computed Tomography (CBCT) allows for real-time visualization with CT imaging. Additionally, CBCT uses an open gantry and provides flexibility to the operator with needle positioning allowing for high accuracy rates even in technically challenging conditions [11]. However, currently, this technique can be labor intensive and slow compared to traditional CT scanners.

Electromagnetic tracking systems (EMTS) use electromagnetic navigation (EMN), an established method to improve accuracy using 3D spatial navigation information [12, 13]. These systems have the ability to fuse several imaging modalities (CT, PT, MRI, or US), thus combining the benefits of each of these modalities to optimize visualization during interventional procedures and providing the ability to approach lesions that were not well visualized on conventional imaging [14–18]. Given the increasing central role that imaging plays in the early detection and diagnosis of a variety of malignancies including lung cancer, colon cancer, and renal cell cancer, [19–21] these systems are emerging synergistically to allow for potential early detection initiatives and to provide support for precisely targeted lesions for diagnostic procedures and minimally invasive treatments.

We connected this investigational EMNTS system to patients who were having CT-guided biopsies to determine the accuracy of this navigational system. To our knowledge, this is the only EMTNS that can generate a 3D fully quantified anatomic map of the target and its surrounding vessels and structures from one preprocedural CT or MRI scan and plan a trajectory to the target lesion clear of vital anatomies. This small pilot study aims to assess the feasibility and accuracy of a 3D quantitative computer aided navigation system, for thoracic and abdominal biopsies as well as interventional oncologic procedures.

## 2. Materials and Methods

### 2.1. 3D Navigation System

**Figure 1:**
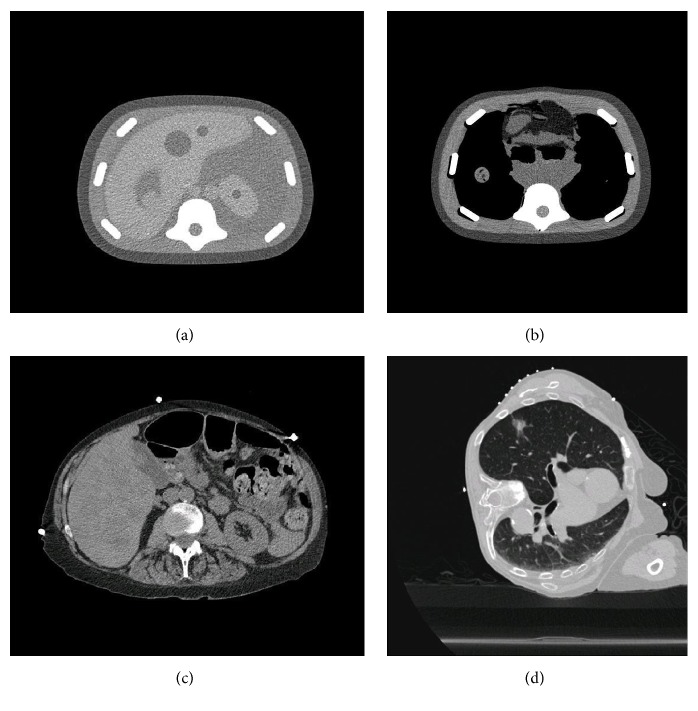
Sample of preprocedural CTs: (a) phantom abdominal scans; (b) phantom thoracic scans; (c) patient abdominal scan; (d) patient thoracic scan.

The quantitative 3D navigation system (IQQA-Guide, EDDA Technology, Inc.) contains an electromagnetic tracking software package and tracks instrument position and orientation in a fully quantified 3D patient-specific anatomy map generated from one preprocedural CT (Figures 1(a)–1(d)) or MRI and its spatial relation to target. All procedures were performed with a Siemens Somatom (Erlangen, Germany) 16-detector CT scanner. CT images were taken with 2 mm thickness with a pitch of 1 : 1.1, 16 × 0.5 mm detector configuration, at 120 peak kilovoltage (kVp) and a variable milliamperage using an effective mAs of 100 mA.

The navigation system has an extendable arm with an electromagnetic field generator attached. The generator, with a working distance of over 40 cm, was positioned facing the intervention area, (patient). CIVCO (Coralville, Iowa) eTRAX coaxial needle system (for liver biopsies) and CIVCO general purpose sensor together with virtuTRAX navigator (for lung biopsies and ablations) were used to acquire position and orientation tracking information of the needle. Tracked fiducial markers were placed on the patient. The electromagnetic- (EM-) tracked fiducial markers allow the “patient-space” to be registered with the 3D patient-specific anatomic map (“image-space”). This registration together with the EM tracking information from the sensor in the eTRAX/virtuTRAX allows the position and orientation of the instrument relative to the 3D anatomic map to be computed and displayed. The patient's registration can be updated with additional intraprocedural scans as needed.

### 2.2. Phantom Experiment

**Figure 2:**
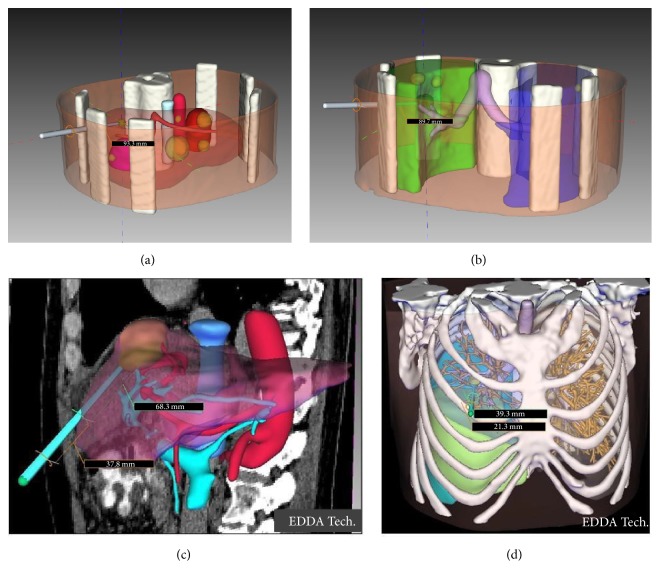
Fully quantified 3D anatomy generated from the CT scans with interactively planned and real-time depth. (a) Abdominal scan in phantom (b) Thoracic scan in phantom (c-d) 3D anatomic map in patients.

An abdominal phantom and a thoracic phantom (CIRS Inc. Norfolk, VA, USA) each with 6 small lesions were used in this study. Preprocedural CT images were sent over the hospital networked to a 3D quantitative navigation system (IQQA-Guide, EDDA technology, Inc., Princeton, NJ). Quantified 3D anatomic renderings of the liver and the lung inclusive of anatomic features (lesions, ducts, skin, bone, etc.) were then generated using the navigation system (Figures 2(a)–2(d)), [22]. Accuracy of the 3D anatomic map registration (i.e., image registration between the initial CT from which the 3D anatomic map was generated and the final needle tip position based on the coregistration of anatomic landmarks such as the carina or major vascular structures and fiducial markers placed on the skin) was determined using final needle tip position on the final conformational CT compared to the expected location of the needle tip based on the 3D model. Initial CT images were performed at the time of the procedure not prior to the procedure.

All procedures with this system were performed by a radiologist with more than 15 years of experience in image guided intervention. The radiologist used either a one-pass or a two-pass method of accessing the specific lesion. In the one-pass needle placement, the physician inserted the needle directly to target using the quantitative 3D navigation system as a guide for appropriate needle positioning. A final conformational CT was performed, to determine final needle positioning; however, no additional imaging was performed during the needle placement to assist with the guidance. For the two-pass needle placement method, the radiologist inserted the needle part of the way to the target using the navigation system as guidance. Then, an intraprocedural CT scan was performed, confirming the location of the tip of the needle. The intraprocedural CT scan was used to update the “patient-space” with “image-space” alignment. The radiologist then inserted the needle to the target using the 3D navigational system and a final conformational CT scan was performed.

### 2.3. Feasibility in Patients

From January 2014 to August 2014, patients with preprocedural CT imaging who were scheduled for a CT-guided biopsy or ablation of the liver or lung were recruited for participation in this IRB approved prospective pilot study. A total of 21 patients were included in this part of the study. During this part of the study the interventional radiologist did not use the navigational system. The system was used passively; that is, the guidance system was not shown to the radiologist during the procedure, and the radiologist was required to perform the procedure as he normally would. The navigational system collected all data and was reviewed for accuracy after the procedure was complete.

### 2.4. Biopsy Procedure

The patient or phantom was positioned on the CT table. All biopsy patients received sedation specifically titrated to moderate sedation using intravenous versed and fentanyl. Both ablation patients received general anesthesia. Five EM-tracked fiducial marks were placed on the phantom or patient in order to create the “patient-space.” In patients, the fiducial markers were placed on the patients' skin outside of the sterile field. An initial CT exam was then performed as part of standard of care and the 3D anatomic map was generated from this dataset. The procedure proceeded as was planned by the radiologist. The radiologist adjusted the needles as was his practice and performed CT scans to verify the needle trajectory and lesion position for targeting purposes. During the biopsy procedure, the navigational system was monitored in order to determine the registration accuracy of the needle tracked comparing the 3D anatomic map and the actual path as seen in the CT images. This was specifically performed by comparing the actual needle tip position based on CT with the computer generated 3D map anticipated needle tip position. The interventional radiologist specifically looked at the actual needle path confirmed by CT as part of the standard of care. The radiologist was not allowed to see the 3D navigational system. With each of the CT scans taken during the procedure, the navigation system registration was updated allowing for continued refinement of needle position.

### 2.5. Accuracy and Workflow Fitness

**Figure 3:**
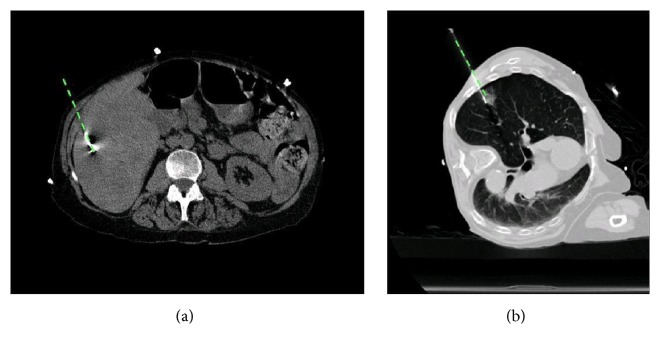
Sample of final confirmation CTs showing the actual needle positions and needle positions projected by the navigation system (dashed lines). (a) Patient abdominal scan; (b) patient thoracic scan.

Accuracy of the system was defined as the distance between the final needle tip position on the conformational CT scan and the anticipated needle tip position predicted by the navigation system (Figures 3(a) and 3(b)). Workflow of the system was also evaluated by the interventional radiologist. Setup time for the system and each of the additional steps necessary to use the navigation system were noted and the time to complete each step was recorded.

### 2.6. Statistical Analysis

All statistical analysis was performed using Graphpad Prism Version 6.0f (La Jolla, CA). All data was analyzed using two-tailed Student's *t*-test. Statistical significance was set at *p* < 0.05.

## 3. Results

### 3.1. Evaluation in Phantoms

In the phantom experiment, a total of 6 thoracic lesions and 6 liver lesions were biopsied with the guidance of the navigation system. The average lesion diameter was 13.2 ± 7.0 mm (SD) (range 4.8 mm–29.8 mm) and the mean distance to the lesions from the surface along the planned path was 76.8 ± 21.3 mm (SD) (range 43.5 mm–121.4 mm). The mean needle placement accuracy in the liver was 2.0 ± 0.7 mm (SD) for one-pass procedures and 2.8 ± 1.1 mm (SD) for two passes and the average procedure time was 9.9 ± 0.2 (SD) minutes (range 9.55−10.1 minutes) and 11.9 ± 0.3 (SD) minutes (range 11.8−12.4), respectively. In thoracic procedures, the mean needle placement accuracy was 2.7 ± 1.7 mm (SD) and 3.0 ± 1.4 mm (SD) for one and two passes, respectively. The average time of navigated needle placement was 8.8 minutes ± 1.1 (SD) (range 6.6–9.8 minutes) for one-pass procedures and 12.8 minutes ± 1.2 (SD) (range 10.8–13.8 minutes) for the two-pass method. The average 3D anatomic map registration error was 1.79 mm.

### 3.2. Clinical Application of Navigation System

In the clinical application segment of this study, a total of 21 patients consented to participate in this study. The average patient was 63.8 years of age (17–85); 68.2% of patients were female; see Table 1. Fifteen procedures were lung biopsy; 5 were targeted liver biopsies; and 2 were lung neoplasm ablations.

The average diameter of targets was 14.3 mm ± 6.7 (7.4–32 mm) SD with a mean distance from skin of 63.1 ± 25.2 mm SD (range 28.7–109.7) (Table 2). Eighteen-gauge biopsy needles were used for lung biopsy, while 16-gauge needles were used liver biopsy. Both lung ablations were performed with a 13-gauge cryoablation probe. The mean accuracy of the 3D navigation system in this passive study was 4.6 mm ± 3.1 (SD) (range 0.88–14.29). Sixteen of the 22 procedures (72.7%) had an accuracy less than 5 mm. There was no significant difference in accuracy between body parts or types of the procedures in this trial (*p* = 0.0802) or the distance to target from the skin surface (*p* = 0.2859). Average time of the system setup was 3.1 minutes.

## 4. Discussion

The results from this study suggest that (1) this 3D navigational system was able to obtain highly accurate needle placement both in phantoms and in patient's procedures; (2) the use of this system is feasible within the existing workflow for interventional procedures.

The needle placement accuracy was evaluated in multiple scenarios. First, in the controlled environment of a phantom study, in this scenario, we were able to document final needle position accuracy of 2.3 ± 1.2 mm. Since a phantom procedure is an idealized situation, we extended these experiments to use in patients. In these cases, the device was used in a passive manner. That is the radiologist performed, the procedure as standard of care without the ability to use or see the 3D navigational system. Accuracy of final needle position was determined by comparing where the 3D navigational system indicated the final needle position and where the CT images indicated the final needle position. This was done to avoid any potential adverse events in patients.

The final aspect of the study was to evaluate this 3D navigational system in clinical scenarios. In addition to determining if this system can generate sufficiently high accuracy in needle position the system must be easily integrated into the clinical workflow. In this study, we measured the time of each part of the procedures. The use of this 3D navigational system added an average of 3.1 minutes. From our experience, using this system, we are confident that adding this system into the clinical workflow should be easily done in an imaging suite as prior EMN image fusion studies have suggested [14–18]. Of interest the degree of variance of the final needle tip position observed with the two-pass method was greater than the degree of variance observed with a one-pass method. There was no clear reason for this difference which was not statistically significantly different.

The navigation system in this study differs from others by providing 3D fully quantified anatomies, making the physician aware of the surrounding structures and facilitating a needle path planning and placement with an intuitive approach. Navigation systems like the one used in this study fuse different 3D imaging modalities (CT, MR, and PET), from previously taken reference datasets to create a 3D workspace. When the target is not well visualized under the 3D working data set (usually US or CT) but had been previously seen, both the 3D working and reference data sets can be superimposed and aligned to create a new 3D working data set in which the electromagnetic needle can be tracked in a real-time multiplanar display. This has shown potential utility for target lesions with FDG avidity in biopsy procedures [15].

Additionally, particularly for ablation treatments, where the delineation of the ablation zone and lesion is more difficult to see during intraprocedural imaging, having the pretreatment image fused with an intraprocedural scan may help to more effectively determine treatment margins. This may translate to better accuracy and treatment outcomes for noninvasive image guided therapies such as cryoablation, radiofrequency ablation (RFA), and microwave ablation (MWA) [23].

The results of this study compare favorably to other studies using similar methodologies. For example, Wallach et al. [24] observed a target positioning error of 4.6 ± 1.2 mm for liver lesions comparing free hand placement to aiming device-navigation. This is similar to the results we found in our phantom studies. This study goes further in that it shows that this system can be translated to clinical use and in both liver and lung procedures with similar (4.6 ± 3.1 mm) accuracy.

There are several limitations to this study. This was a pilot study with a small number of patients and future randomized controlled trials in which the trajectory planned by the 3D navigation system is utilized will be necessary to give a clear understanding of the utility and benefits of a 3D electromagnetic navigation device with image fusion capabilities. Although respiratory motion was incorporated into the navigational system, the use of these in patients with erratic respiratory cycles has not been tested. Needle bending was not evaluated in this study; however, the needles used in this study are of a gauge where there is less risk of significant needle bending. The use of these needles also adds an additional bias; the potential for more significant and important needle bending could occur with thinner needle gauges.

Tissue deformation was not considered in this study. This is a common issue in solid organs but is obviated in the extreme scenario of an intraprocedural pneumothorax. Most common tissue deformation refers to the change in shape and position of an organ or target lesion after the introduction of a needle. Despite these limitations and potentially confounding factors this study shows that this 3D electromagnetic navigational system is able to attain extremely high accuracy level of needle placement in both phantoms and humans, in the clinical scenario or liver biopsy, lung biopsy, and lung ablation. This study suggests that this system could be used in many clinical scenarios with limited impact of clinical workflow and potentially with improved clinical outcomes. Future studies using this system to actively guide biopsy and ablation procedures are necessary to fully test this system.

## Figures and Tables

**Table 1:** Patient demographics.

	Patient demographics (*n* = 21)
Mean age, years	63.8 (17–85)
Gender	
Male, *n*	6
Female, *n*	15
Lung biopsy, *n*	15
Lung cryoablation, *n*	2
Liver biopsy, *n*	5

**Table 2:** Interventional procedure details.

	Interventional procedures (*n* = 22)
Mean target diameter (mm)	14.3 ± 6.7 (7.4–32.0)
Mean target distance from skin (mm)	63.1 ± 25.2 (28.7–109.7)
